# Ability of the Nutri-Score front-of-pack nutrition label to discriminate the nutritional quality of foods in the German food market and consistency with nutritional recommendations

**DOI:** 10.1186/s13690-019-0357-x

**Published:** 2019-06-14

**Authors:** Fabien Szabo de Edelenyi, Manon Egnell, Pilar Galan, Nathalie Druesne-Pecollo, Serge Hercberg, Chantal Julia

**Affiliations:** 1 Université Paris 13, Equipe de Recherche en Epidémiologie Nutritionnelle (EREN), Centre de Recherche en Epidémiologie et Statistiques, Inserm (U1153), Inra(U1125), Cnam, COMUE Sorbonne Paris Cité, F-93017 Bobigny, 74 rue Marcel Cachin, F-93017 Bobigny Cedex, France; 2 Département de Santé Publique, Hôpital Avicenne (AP-HP), F-93017 Bobigny, CJ SH France

**Keywords:** Nutri-Score, nutrient profiling system, Nutritional quality, Front-of-pack labelling, Food-based dietary guidelines

## Abstract

**Background:**

There is currently a societal debate in Germany concerning the interest to introduce a comprehensive and simplified nutritional information label on foods. Consumer associations and some manufacturers are supporting the Nutri-Score, a summary, graded, colours-coded front-of-pack label (FoPL) adopted by public health authorities in France, Belgium and Spain. The Nutri-Score is using a Nutrient Profiling System (NPS) to define five different categories of nutritional quality (from ‘Dark green’ associated with the letter A to ‘dark orange’ with an E). The ability of the Nutri-Score to discriminate nutritional quality of foods was demonstrated in the French context. The objectives of this study were to verify its ability to discriminate the nutritional quality of foods and beverages currently present on the market in Germany and its consistency with German Food-Based Dietary Guidelines (FBDG).

**Methods:**

Nutritional composition of 8587 usual foods available on the German market collected from the web-based collaborative project Open Food Facts, were retrieved. Data were collected from 2012 to 2019, with regular updates each time a product is scanned again by a contributor. Distribution of products across the five Nutri-Score categories according to consumer-based food groups was assessed. The ability of the FoPL to discriminate the nutritional quality of foods and beverages was estimated by the number of available colours of the Nutri-Score in each food group and sub-groups.

**Results:**

Overall, the classification of foods according to the Nutri-Score was consistent with German FBDG: foods which consumption is recommended were more favourably classified (e.g. 79.7% of products composed mainly of fruits and vegetables were classified as A or B) than foods which consumption should be limited (e.g. 93.4% of sugary snacks were classified as D or E).

Moreover, we observed that the nutrient profiling system underpinning the Nutri-Score was able to display the variability in nutritional quality of foods within the same food groups, with good discriminating performance (at least three colours represented with the Nutri-Score).

**Conclusions:**

The Nutri-Score label displays a high ability in discriminating nutritional quality of foods across food groups and within a food group in the German market. This element is a key step in the validation process of a front-of-pack label, so that the Nutri-Score is an efficient tool which could help German consumers to make healthier choices.

## Background

**Fig. 1:**
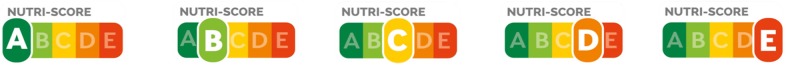
Graphic format of Nutri-Score

Front-of-Pack Labels (FoPLs) and more specifically interpretative FoPLs, giving directly an evaluative assessment of the nutritional quality of foods to consumers, are considered as a cost-effective measure recommended by the World Health Organization as one of the “best buys” measures to prevent Non-Communicable Diseases (NCDs) [1, 2]. In this context, in order to tackle the increasing burden of diet-related NCDs, French government adopted in 2017 the Nutri-Score [3], a summary, graded, colour-coded FoPL with twin objectives: 1) to provide a helpful guidance for consumers towards healthier food choices at the point of purchase, as it delivers at-a-glance simplified nutritional information, and 2) to incentivize manufacturers to reformulate their products towards healthier composition, which would be materialized on the FoPL [4, 5]. The Nutri-Score was developed by independent French researchers and was chosen by French public health authorities as it was supported by a strong scientific background [6]. This FoPL relies on the computation of a score substantially based on the United Kingdom Food Standards Agency Nutrient Profiling System (FSA-NPS), which was developed to regulate television advertising to children [7–9]. The FSA score is computed taking into account the nutrient content per 100 g for foods [6]. The algorithm allocates positive points (0–10) for unfavourable elements including energy (kJ), total sugars (g), saturated fatty acids (g) and sodium(mg), and negative points (0–5) for favourable elements including fruits/vegetables/pulses/nuts (%), fibres (g) and proteins (g). The sum from positive points (0 to + 40 points) and negative points (0 to-15 points) is computed, yielding a global score ranging from − 15 for the healthiest foods to + 40 for less healthy foods. From this overall score, five categories of nutritional quality are derived, defining the categories for the Nutri-Score, ranging from ‘dark green’ to ‘dark orange’ (Fig. 1). Letters (A to E) were added to colours in order to improve the readability of the label, in particular for the colour-blind. The entire scale appears on the label, with the letter/colour corresponding to the product’s nutritional quality enlarged. Though the FSA-NPS is based on an across-the-board approach, some marginal adaptations were pointed as necessary in a report from the French Agency for Food, Environmental and Occupational Health and Safety, ANSES [10] to improve consistency with nutritional recommendations for all categories of foods. To correct these limitations, the FSA nutrient profiling algorithm was slightly modified for cheeses, added fats and beverages by the French High Council of Public Health (FSAm-NSP) [11].

Between the initial proposal in 2013 [12] and the selection of the Nutri-Score by the French public health authorities in 2017, multiple scientific studies on the Nutri-Score were conducted [6] pertaining to both the validation of the FSA-NPS underpinning the system and the validation of its visual appearance (graphical format). Most of these studies were performed in the French context, questioning the potential generalization of the positive results of the Nutri-Score in France to other different cultural contexts with their own food markets.

This question is particularly topical in Germany where discussions are currently ongoing concerning the possible adoption of a FoPL by the government [13]. Different consumer associations [14] and some manufacturers [15] have declared their support to the Nutri-Score scheme; however issues have been raised concerning the suitability of the Nutri-Score in the German context in terms of graphical design and nutrient profiling system [16]. Regarding the relevance of the Nutri-Score graphical format for German consumers, a recent international study provided scientific evidence [17]. This international comparative experimental study aimed to compare the ability of five FoPLs [Nutri-Score, Australian Health Star Rating system (HSR), UK Multiple Traffic Lights (MTL), Chilean Warning labels and Reference Intakes (RIs) endorsed by manufacturers] to help consumers to understand the nutritional quality of different types of foods within different categories, in 12 countries including Germany. Results showed that the Nutri-Score performed best in all countries to help consumers correctly rank products according to their nutritional quality. This favourable effect was also found in the sample of the 1000 German consumers participating to the study.

If the graphical design of the Nutri-Score seems appropriate to the German socio-cultural context, the relevance of the FSA-NPS underlying the 5 categories of the Nutri-Score for the food German market, as it was demonstrated in the French food market, requires further investigation. So, it appears of importance to assess how the Nutri-Score classifies foods in the German market and whether this classification aligns with the German food-based dietary guidelines (FBDG).

Thus, the objectives of this study were 1) to test the ability of the Nutri-Score to discriminate the nutritional quality of foods and beverages currently available on the German market using a wide food database including branded products, and 2) to investigate the consistency between the classification of branded foods by the Nutri-Score and the German FBDG.

## Methods

### Food composition database

Food composition data concerning German foods was retrieved from the Open Food Facts project database, an international collaborative web project based on a wiki-like interface gathering food composition data based on available back-of-pack labelling of products (https://de.openfoodfacts.org/). Using crowdsourcing to collect food composition data of the food supply, specific data are collected by volunteer contributors including information about ingredients (including percentages of fruits and vegetable, legumes and nuts which are required for the computation of the Nutri-Score) and nutrition facts (including energy and mandatory nutrient-content per 100 g: sugars, saturated fatty acids and sodium which are also used for the computation of the Nutri-Score) from foods purchased in stores. The collected data are available freely as an open data source. We retrieved specific data on foods sold in Germany from national brands, store brands and discount brands. The database extract date for this analysis was February 12th, 2019. Controlled quality procedures included manual check based on outliers detection (over P99) on individual variables used in the calculation of the Nutri-Score in addition to controls already done at the OpenFoodFacts database level. Moreover, we also manually checked products with a mismatch between the energy calculated using carbohydrates, lipids and proteins contents and the energy variable in the database. Potential errors were corrected when possible using images available on OpenFoodFacts website. Otherwise the products were removed from analysis. Data were collected from 2012 to 2019, with regular updates each time a product is scanned again by a contributor.

### Food classification

Foods were categorized using a consumer’s point of view, grouping foods with similar use and with distinct nutritional characteristics. Main food groups included ‘Products containing mainly fruits and vegetables’, ‘Cereals and potatoes’, ‘Meat, Fish and Eggs’, ‘Milk and dairy products’, ‘Fats and sauces’, ‘Composite foods’, ‘Sugary snacks’, ‘Salty snacks’ and ‘Beverages’. Within each food group, sub-groups were identified (e.g. in the ‘Cereals and potatoes’ main group, sub-categories included ‘Bread’, ‘Cereals’, ‘Legumes’, ‘Potatoes’ and ‘Breakfast cereals’). Each food was categorized in a single food group and sub-group. Herbs and spices, or special use products were excluded from the analysis, as they are not included in the perimeter of the Nutri-Score. Foods for which the nutritional composition was incomplete for the computation of the Nutri-Score were also excluded (*N* = 2781), as well as foods with missing food group (*N* = 3289).

### Statistical analyses

The distribution of the overall FSAm-NSP was computed in the different food groups, and displayed using boxplots, highlighting the median, 25th and 75th percentiles of the distribution. Distribution of foods and beverages in the different categories of the Nutri-Score was also computed. Ability of the FoPL to discriminate nutritional quality of foods and beverages was estimated by the number of available colours in each group and sub-groups. When three or more colours were available in a food group, the discriminating ability of the Nutri-Score was considered good, in a pragmatic approach.

The consistency of the food classification using the Nutri-Score with the German food-based dietary guidelines (http://www.fao.org/nutrition/education/food-dietary-guidelines/regions/countries/germany/en/) was assessed by comparing for each food group the distribution of foods in the different Nutri-Score categories with the recommended consumption frequency of the group. Thus, food groups which consumption is encouraged by the dietary guidelines should be classified “favourable” by the Nutri-Score (i.e. A / dark green or B / green) while groups which consumption has to be limited should be classified “unfavourable” by the Nutri-Score (i.e. D / orange or E/dark orange). German dietary guidelines are available as supplemental material.

## Results

Concerning the German market, manufactured items with complete available data for the computation of the FSAm-NSP score in the Open Food Facts database were included in the analyses, corresponding to 8587 foods and beverages: 527 products composed mainly of fruits and vegetables, 1396 bread and cereal products, 688 meat, fish and eggs products, 1875 milk and dairy products, 619 fats and sauces, 452 composite foods, 1745 sugary snacks, 413 salty snacks, and 872 beverages. Overall, the mean FSAm-NSP score was 9.6 + 9.6 points.

**Fig. 2:**
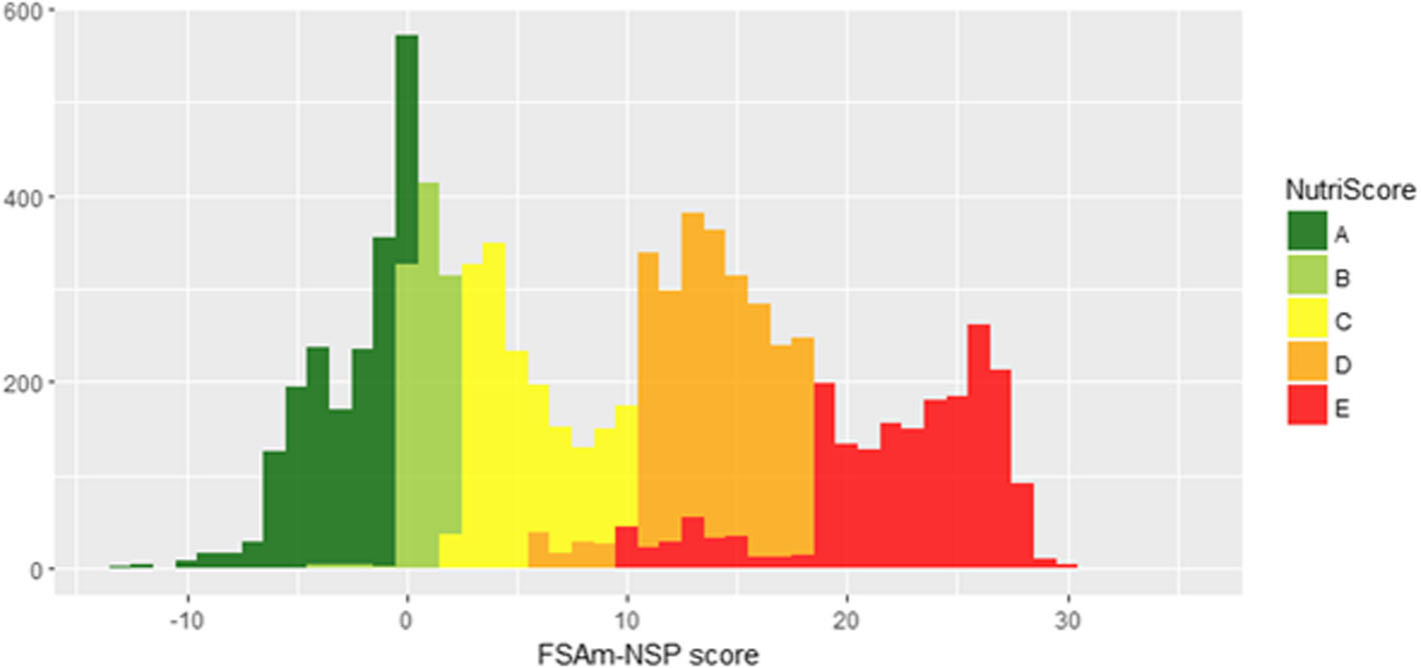
Distribution of the FSAm-NSP score

The overall distribution of the FSAm-NSP score with the different Nutri-Score categories is presented in Fig. 2 Overall, 18.9% of foods were classified in the A category; 12.1% as B; 18.5% as C; 27.5% as D; and 23.0% E.

**Fig. 3:**
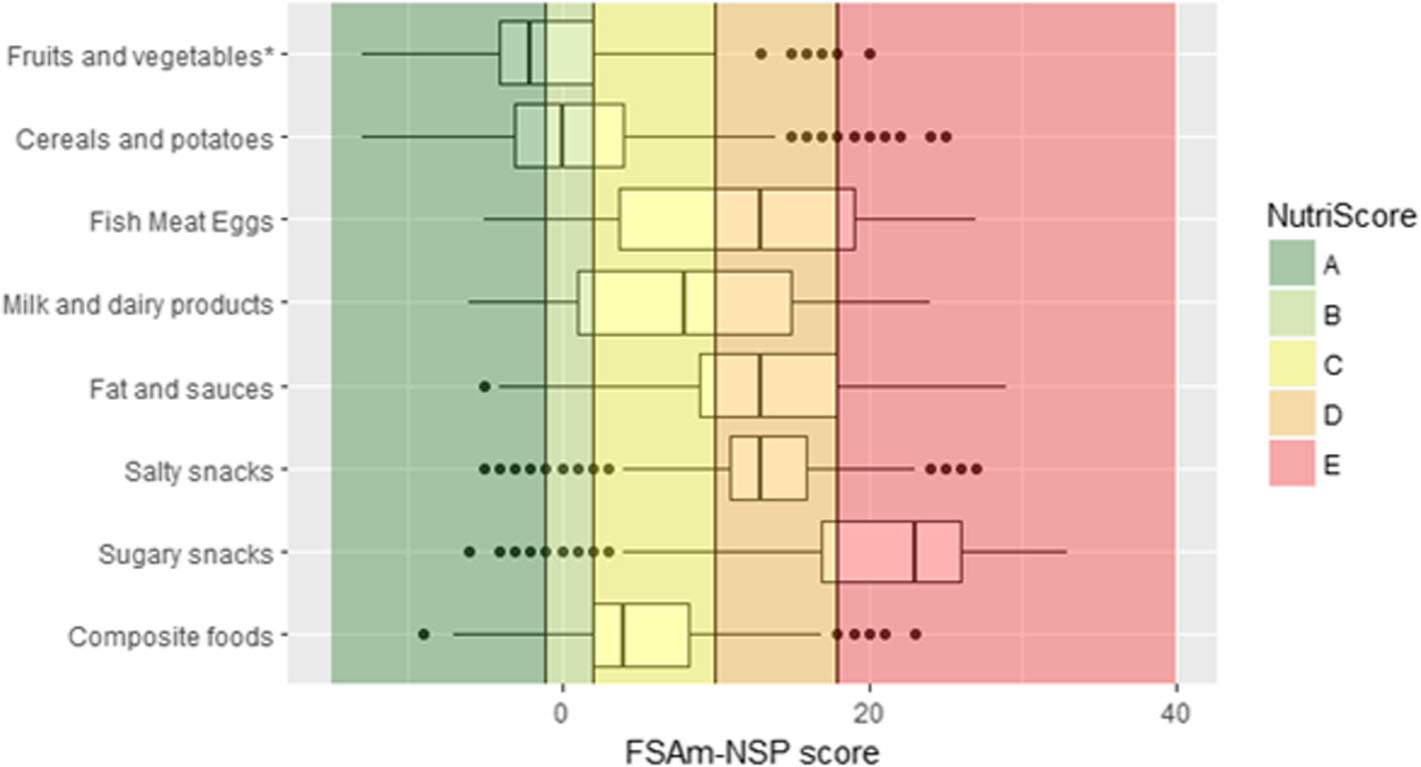
Distribution of the FSAm-NSP score for solid foods.Vertical lines represent the cut-offs of the 5-category Nutri-Score. The boundary of the box nearest to the left indicates the 25th percentile, the line within the box marks the median, and the boundary of the box furthest from the left indicates the 75th percentile. Whiskers (error bars) left and right of the box indicate the lower limit (25th percentile - 1.5 * (Inter-quartile range) and the upper limit (75th percentile + 1.5 * (Inter-quartile range)). The circles are individual outlier points. *Products containing mainly fruits and vegetables

**Fig. 4:**
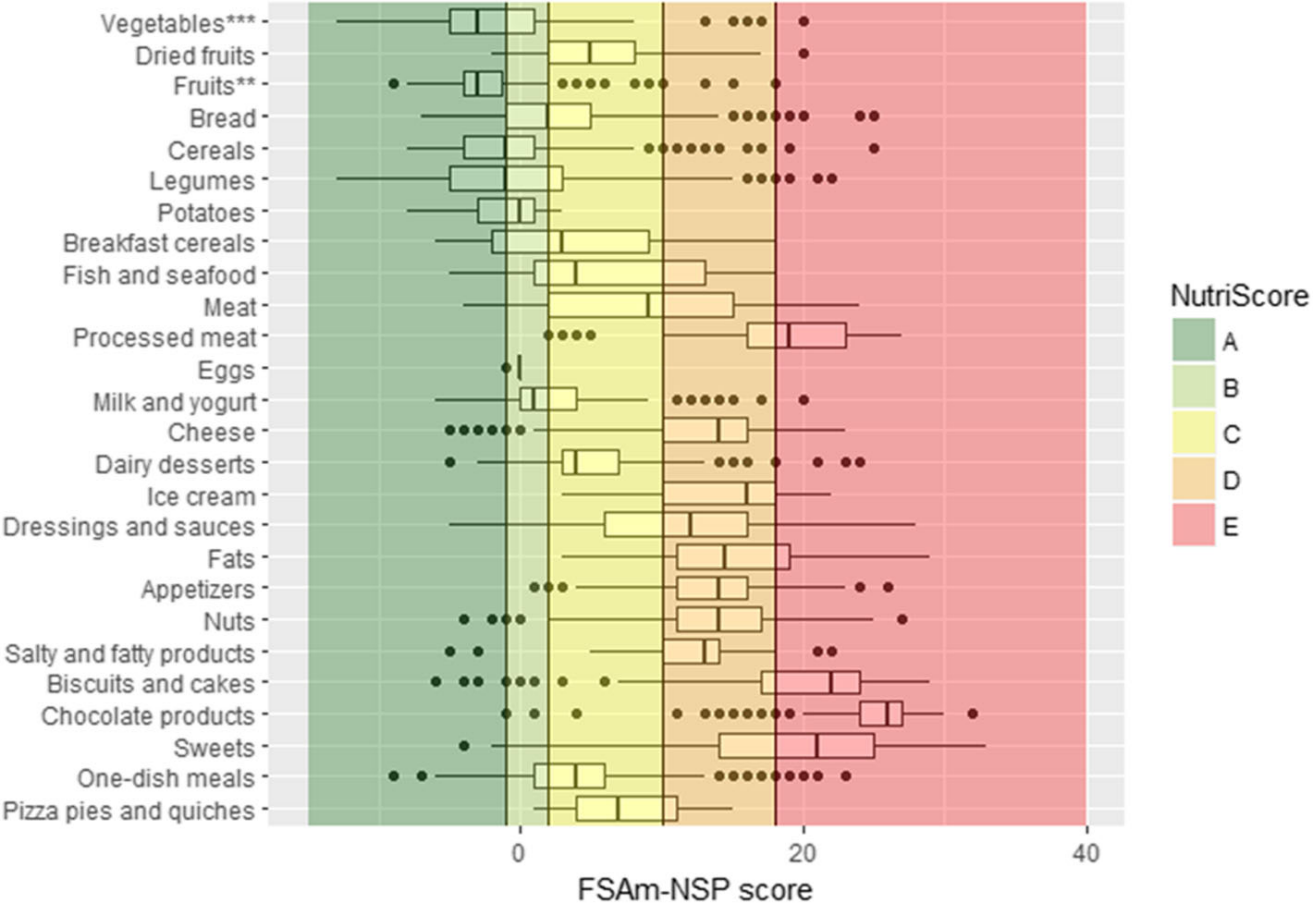
Distribution of the FSAm-NSP score for solid foods in sub-groups containing more than 20 items. Vertical lines represent the cut-offs of the 5-category Nutri-Score. The boundary of the box nearest to the left indicates the 25th percentile, the line within the box marks the median, and the boundary of the box furthest from the left indicates the 75th percentile. Whiskers (error bars) left and right of the box indicate the lower limit (25th percentile - 1.5 * (Inter-quartile range) and the upper limit (75th percentile + 1.5 * (Inter-quartile range)). The circles are individual outlier points. ** Fruits based products .*** Vegetables based products

**Fig. 5:**
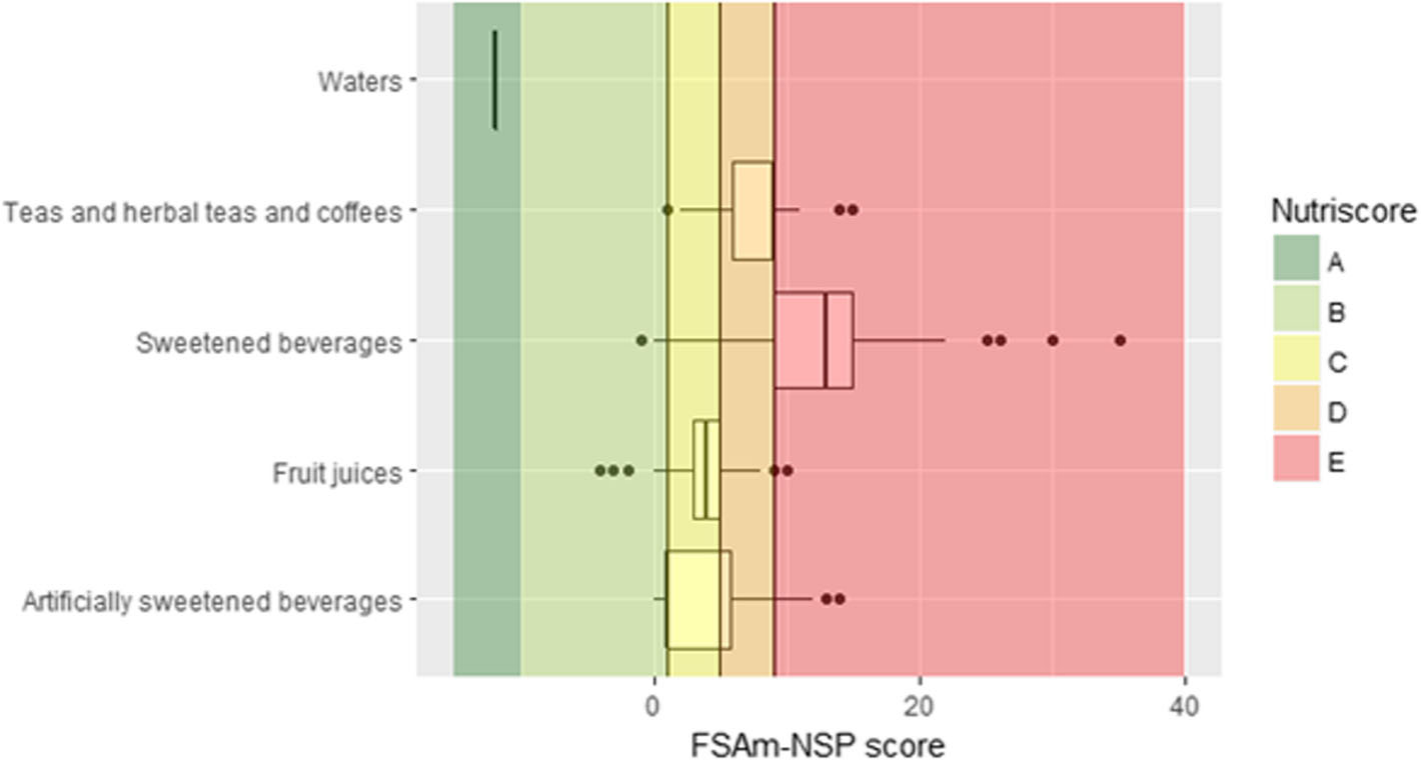
Distribution of the FSAm-NSP score for beverages. Vertical lines represent the cut-offs of the 5-category Nutriscore. The boundary of the box nearest to the left indicates the 25th percentile, the line within the box marks the median, and the boundary of the box furthest from the left indicates the 75th percentile. Whiskers (error bars) left and right of the box indicate the lower limit (25th percentile - 1.5 * (Inter-quartile range) and the upper limit (75th percentile + 1.5 * (Inter-quartile range)). The circles are individual outlier points. By definition, only water is classified as A and is shown at the top of the plot

The distribution of the FSAm-NSP score with the different Nutri-Score categories within each food group is displayed in Fig. 3 for all solid foods, in Fig. 4 for sub-groups of solid foods containing at least 20 items, and in Fig. 5 for the beverages.

The distribution of the Nutri-Score within the different food groups and sub-groups is displayed in Table 1. A total of 79.7% of products from “fruits and vegetables”, 69.3% of products from “Cereals and potatoes” were classified as dark green (A) or green (B), while 93.4% of products from “Sugary snacks” were classified as orange (D) or dark orange (E). Among beverages, while a majority of fruit juices were classified as C (70.1%), soft drinks were classified as E.

**Table 1:** Distribution of the Nutri-Score within the different food groups

		A	B	C	D	E	Total
Fruits and vegetable*		**323(61.4%)**	**97(18.4%)**	**95(18.0%)**	**10(1.9%)**	**2(0.4%)**	**527**
Vegetables***		189(64.5%)	75(25.6%)	23(7.8%)	5(1.7%)	1(0.3%)	293
Dried fruits		4(5.6%)	15(20.8%)	50(69.4%)	2(2.8%)	1(1.4%)	72
Fruits**		130(80.2%)	7(4.3%)	22(13.6%)	3(1.9%)	0(0%)	162
**Cereals and potatoes**		**689(49.4%)**	**278(19.9%)**	**264(18.9%)**	**146(10.5%)**	**19(1.4%)**	**1396**
Bread		103(30.9%)	76(22.8%)	100(30.0%)	49(14.7%)	5(1.5%)	333
Cereals		377(61.9%)	132(21.7%)	75(12.3%)	23(3.8%)	2(0.3%)	609
Legumes		109(60.2%)	26(14.4%)	10(5.5%)	24(13.3%)	12(6.6%)	181
Potatoes		10(43.5%)	10(43.5%)	3(13.0%)	0(0%)	0(0%)	23
Breakfast cereals		90(36.0%)	34(13.6%)	76(30.4%)	50(20.0%)	0(0%)	250
**Fish Meat Eggs**		**53(7.7%)**	**97(14.1%)**	**92(13.4%)**	**259(37.6%)**	**187(27.2%)**	**688**
Fish and seafood		34(15.5%)	41(18.6%)	51(23.2%)	94(42.7%)	0(0%)	220
Meat		17(13.6%)	19(15.2%)	28(22.4%)	36(28.8%)	25(20%)	125
Processed meat		0(0%)	1(0.3%)	12(3.9%)	129(42.4%)	162(53.3%)	304
Eggs		2(5.3%)	36(94.7%)	0(0%)	0(0%)	0(0%)	38
Offals		0(0%)	0(0%)	1(100%)	0(0%)	0(0%)	1
**Milk and dairy products**		**241(12.9%)**	**339(18.1%)**	**440(23.5%)**	**795(42.4%)**	**60(3.2%)**	**1875**
Milk and yogurt		127(18.7%)	268(39.5%)	209(30.8%)	73(10.8%)	2(0.3%)	679
Cheese		108(11.7%)	50(5.4%)	109(11.8%)	625(67.7%)	31(3.4%)	923
Dairy desserts		6(4.7%)	21(16.4%)	85(66.4%)	13(10.2%)	3(2.3%)	128
Ice cream		0(0%)	0(0%)	37(25.5%)	84(57.9%)	24(16.6%)	145
**Fat and sauces**		**13(2.1%)**	**17(2.7%)**	**165(26.7%)**	**302(48.8%)**	**122(19.7%)**	**619**
Dressings and sauces		13(3.2%)	17(4.2%)	138(34.2%)	185(45.9%)	50(12.4%)	403
Fats		0(0%)	0(0%)	27(12.5%)	117(54.2%)	72(33.3%)	216
**Salty snacks**		**6(1.5%)**	**8(1.9%)**	**80(19.4%)**	**262(63.4%)**	**57(13.8%)**	**413**
Appetizers		0(0%)	3(1.5%)	36(18.2%)	139(70.2%)	20(10.1%)	198
Nuts		4(2.5%)	5(3.2%)	30(19.0%)	87(55.1%)	32(20.3%)	158
Salty and fatty products		2(3.5%)	0(0%)	14(24.6%)	36(63.2%)	5(8.8%)	57
**Sugary snacks**		**13(0.7%)**	**40(2.3%)**	**62(3.6%)**	**386(22.1%)**	**1244(71.3%)**	**1745**
Biscuits and cakes		5(1.1%)	2(0.5%)	9(2%)	125(28.4%)	299(68%)	440
Chocolate products		1(0.2%)	2(0.4%)	1(0.2%)	34(6.0%)	533(93.3%)	571
Sweets		7(1.0%)	36(5.0%)	50(6.9%)	224(31.1%)	403(56.0%)	720
Pastries		0(0%)	0(0%)	2(14.3%)	3(21.4%)	9(64.3%)	14
**Composite foods**		**39(8.6%)**	**97(21.5%)**	**217(48.0%)**	**94(20.8%)**	**5(1.1%)**	**452**
One-dish meals		39(11.4%)	88(25.7%)	164(48.0%)	46(13.5%)	5(1.5%)	342
Pizza pies and quiche		0(0%)	9(8.5%)	53(50.0%)	44(41.5%)	0(0%)	106
Sandwiches		0(0%)	0(0%)	0(0%)	4(100%)	0(0%)	4
**Beverages**		**245(28.1%)**	**63(7.2%)**	**173(19.8%)**	**111(12.7%)**	**280(32.1%)**	**872**
Waters		245(100%)	0(0%)	0(0%)	0(0%)	0(0%)	245
Teas and herbal teas and coffees		0(0%)	2(9.1%)	2(9.1%)	13(59.1%)	5(22.7%)	22
Fruit juices		0(0%)	15(7.5%)	141(70.1%)	21(10.4%)	24(11.9%)	201
Fruit nectars		0(0%)	0(0%)	0(0%)	2(16.7%)	10(83.3%)	12
Artificially sweetened beverages		0(0%)	33(53.2%)	13(21%)	9(14.5%)	7(11.3%)	62
Sweetened beverages		0(0%)	13(3.9%)	17(5.2%)	66(20%)	234(70.9%)	330
**Sum**		**1622(18.9%)**	**1036(12.1%)**	**1588(18.5%)**	**2365(27.5%)**	**1976(23.0%)**	**8587**

*Fruits or vegetable based products

**Fruits based products

***Vegetables based products

For foods: the FSAm-NPS score ranges from − 15 to − 1 points for the A category, from 0 to 2 for the B category, from 3 to 10 for the C category, from 11 to 18 for the D category, and 19 to 40 points for the E category.

For beverages: A corresponds to mineral waters exclusively. The FSAm-NPS score ranges from − 15 to 1 point for the B category, from 2 to 5 for the C category, from 6 to 9 for the D category, and from 10 to 40 points for the E category

**Fig. 6:**
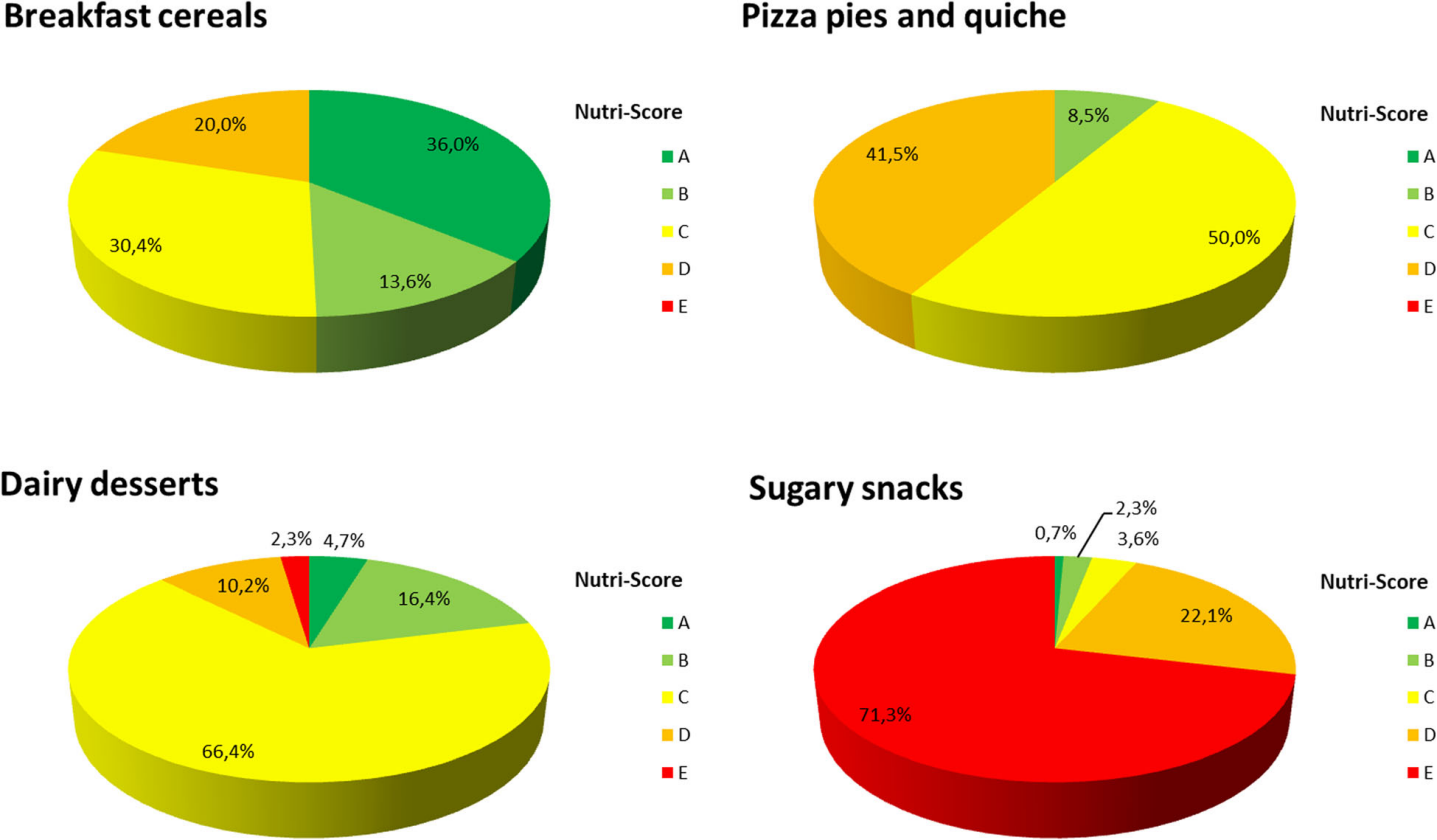
Pie charts of Nutri-Score distributions in four food groups: Breakfast cereals, Pizza pies and quiche, Dairy desserts and Sugary snacks

Moreover, within almost each food group, differences in the nutritional quality of products between sub-groups were grasped by the Nutri-Score classification, with high discriminating ability (at least three colours represented as defined in the methods section). Thus, for example, within the “Milk and dairy products” sub-group, foods from the sub-group “Milk and yogurt” were mainly classified as products with higher nutritional quality – between dark green (A) and yellow (C) – than foods from “Ice creams” mainly categorized between yellow (C) and dark orange (D). To illustrate the results from Table 1, pie charts for 4 key food groups (Breakfast cereals, Pizza pies and quiche, Dairy desserts and Sugary snacks) are shown in Fig. 6.

## Discussion

In the present study, results showed that the Nutri-Score, based on the FSA nutrient profiling system adapted by the HCSP, is an efficient tool to discriminate products (solid foods and beverages) across and within food groups and sub-groups, with at least three categories of Nutri-Score represented. Overall, the classification of the different food groups in the Nutri-Score displayed a high consistency with German nutritional recommendations [18, 19]. Indeed, foods which consumption is recommended (e.g. 79.7% of products composed mainly of fruits and vegetables classified as A or B) were more favourably classified than foods which consumption should be limited (e.g. 93.4% of sugary snacks classified as D or E). Within a food group, the same discrimination was observed, as foods lower in salt, sugar and fat were better classified. The distribution of the FSAm-NSP score underpinning the Nutri-Score displayed a high variability, confirming its validity for use in the 5-category label Nutri-Score in the context of the German food market.

The discriminating ability of the Nutri-Score is a key element to help consumers making healthier choices at the point of purchases, by displaying with at-a-glance labelling the nutritional quality of products.

These results represent a key step in the validation process of a FoPL, which underlying nutrient profiling system has to be validated upstream in scientific studies. In the theoretical framework of Townsend et al., the classification of foods by the nutrient profiling system against national dietary recommendations is one of the major elements [20]. The findings of the present study specific to the German context are consistent with those investigating the consistency of the score underpinning the Nutri-Score in the French context, using nutritional composition data from different databases (generic foods and branded products) [21–23]. In the French food environment, the classification of foods was overall consistent with French nutritional recommendations (which are very similar to German recommendations) and the discriminating ability of the 5 colours nutrition label (previous graphical format of the Nutri-Score) was similar in France and Germany across food groups, within food groups and to a lower extent for equivalent foods from different brands. Finally, these results in Germany as in France suggest that the use of the FSAm-NSP score associated with the Nutri-Score, while being ‘across-the-board’ from most food items, would support both possible ‘displacement’ and ‘substitution’ strategies, as nutritional quality across food groups, but also within food groups is consistently discriminated.

The main limitation of the study pertains to the use of the Open Food Facts database. Indeed, though the Open Food Facts database collects data from products currently available on the market directly from consumers, we were not able to analyze the representativeness of the sample of foods retrieved, either in terms of number of products or market share. However, our purpose was not to be exhaustive, but rather to test the discriminating ability of the Nutri-Score in real-life situations, for which the Open Food Facts database is sufficiently large to give a consistent evaluation.

## Conclusions

Finally, the Nutri-Score appears as an efficient tool which could help German consumers to discriminate nutritional quality of foods at various levels of details in foods marketed in Germany, whilst avoiding a dichotomous thinking of foods in ‘healthier’ and ‘less healthy’ categories promoting the contention that foods are either ‘all good’ or ‘all bad’. As a result, it would help consumers to be aware of the specific nutritional quality of foods and making healthier choices at the point of purchase. As the graphical format of the Nutri-Score appeared also as the best option in German consumers compared to other formats, overall these results suggest the Nutri-Score would be a valid choice in the German context. The German situation regarding the implementation of the Nutri-Score in German supermarkets would also have a direct impact on other countries, especially on the European food market. Indeed, the adoption of a single front-of-pack nutrition label in the different countries would be particularly important for industrialists and retailers exporting food products from and in Germany.

## Data Availability

Food composition data concerning German foods was retrieved from the Open Food Facts project database (https://de.openfoodfacts.org/). Accessed February 12th 2019.

## Abbreviations

FBDG: Food-Based Dietary Guidelines
FoPL: Front-of-Pack Label
FSA: Food Standards Agency
HCSP: Haut Conseil de la Santé Publique
NPS: Nutrient Profiling System

## References

[1] http://www.euro.who.int/en/health-topics/disease-prevention/nutrition/publications/2018/what-is-the-evidence-on-the-policy-specifications. Accessed February 13 2019.

[2] WHO World Health Organization. Tackling NCDs: ‘best buys’ and other recommended interventions for the prevention and control of noncommunicable diseases. World Health Organization; 2017. 25p.

[3] Journal Officiel de la République Française. JORF n°0257 du 3 Novembre 2017. texte n° 16. Arrêté du 31 octobre 2017 fixant la forme de présentation complémentaire à la déclaration nutritionnelle recommandée par l'Etat en application des articles L. 3232–8 et R. 3232–7 du code de la santé publique. Paris: JORF; 2017. Availablefrom: https://www.legifrance.gouv.fr/eli/arrete/2017/10/31/SSAP1730474A/jo/texte. Accessed February 13^th^ 2019.

[4] Vyth EL, Steenhuis IH, Roodenburg AJ, Brug J, Seidell JC (2010). Front-of-pack nutrition label stimulates healthier product development: a quantitative analysis. IntJBehavNutrPhysAct..

[5] Ni Mhurchu C, Eyles H, Choi Y-H. Effects of a Voluntary Front-of-Pack Nutrition Labelling System on Packaged Food Reformulation: The Health Star Rating System in New Zealand. Nutrients [Internet]. 2017 August [cited 2019 Feb 1];9(8). Available from: https://www.ncbi.nlm.nih.gov/pmc/articles/PMC5579711. Accessed February 13^th^ 2019. 10.3390/nu9080918 PMC5579711 28829380

[6] Julia C, Hercberg S (2017). Development of a new front-of-pack nutrition label in France: the 5-colour Nutri-score. Public Health Panorama.

[7] Rayner, M., Scarborough, P., Stockley, L., and Boxer, A. Nutrient profiles: development of final model. Final Report . Available from: https://www.researchgate.net/publication/266447771_Nutrient_profiles_Development_of_Final_Model_Final_Report. Accessed February 13^th^ 2019.

[8] Rayner, M., Scarborough, P., and Stockley, L. Nutrient profiles: applicability of currently proposed model for uses in relation to promotion of foods in children aged 5–10 and adults. [Internet]. Available from: https://www.researchgate.net/publication/267952402_Nutrient_profiles_Applicability_of_currently_proposed_model_for_uses_in_relation_to_promotion_of_food_to_children_aged_5-10_and_adults. Accessed February 13 2019.

[9] Rayner, M., Scarborough, P., and Lobstein, T. The UK Ofcom nutrient profiling model - defining 'healthy' and 'unhealthy' food and drinks for TV advertising to children. [Internet] Available from: https://www.ndph.ox.ac.uk/cpnp/files/about/uk-ofcom-nutrient-profile-model.pdf.Accessed February 13 2019.

[10] ANSES. Evaluation de la faisabilité du calcul d'un score nutritionnel tel qu'élaboré par Rayner et al. Rapport d'appui scientifique et technique.ANSES :Maison Alfort. Available from: https://www.anses.fr/fr/system/files/DER2014sa0099Ra.pdf. Accessed February 13^th^ 2019.

[11] Haut Conseil de la Santé Publique. Avis relatif à l'information sur la qualité nutritionnelle des produits alimentaires. Paris :HCSP. Availablefrom: http://www.hcsp.fr/Explore.cgi/avisrapportsdomaine?clefr=519. Accessed February 13^th^ 2019.

[12] Hercberg, S. Propositions pour un nouvel élan de la politique nutritionnelle française de santé publique dans le cadre de la stratégie nationale de santé. 1ère partie : mesures concernant la prévention nutritionnelle. 2013. Available from: https://solidarites-sante.gouv.fr/IMG/pdf/rapport_Hercberg_15_11_2013.pdf. Accessed February 13^th^ 2019.

[13] https://www.faz.net/aktuell/wirtschaft/die-lebensmittelampel-ist-hoechst-umstritten-16010920.html. Accessed February 13^th^ 2019.

[14] https://www.euractiv.com/section/agriculture-food/news/consumer-group-urges-germany-to-stop-opposing-colour-coded-nutrition-labeling. Accessed February 13 2019.

[15] http://www.spiegel.de/wirtschaft/nutri-score-warum-kuenftig-einen-naehrwertampel-gibt-a-1252157.html. Accessed February 13^th^ 2019.

[16] https://www.aerztezeitung.de/panorama/ernaehrung/article/980164/nutri-score-kloeckner-noch-nicht-naehrwert-logo-entschieden.html. Accessed February 13^th^ 2019.

[17] Egnell Manon, Talati Zenobia, Hercberg Serge, Pettigrew Simone, Julia Chantal (2018). Objective Understanding of Front-of-Package Nutrition Labels: An International Comparative Experimental Study across 12 Countries. Nutrients.

[18] Jungvogel A, Michel M, Bechthold A (2016). Die lebensmittelbezo-genen Ernährungsempfehlungen der DGE. Ernährungs Umschau.

[19] Oberritter H, Schäbethal K (2013). Rüsten a v. et al. the DGE nutrition circle – presentation and basis of the food-related recommendations from the German nutrition society (DGE). Ernahrungs. Umschau..

[20] Townsend MS (2010). Where is the science? What will it take to show that nutrient profiling systems work?. Am J Clin Nutr.

[21] Julia C, Ducrot P, Peneau S (2015). Discriminating nutritional quality of foods using the 5-color nutrition label in the French food market: consistency with nutritional recommendations. Nutr J.

[22] Julia C, Kesse-Guyot E, Ducrot P (2015). Performance of a five category front-of-pack labelling system - the 5-colour nutrition label - to differentiate nutritional quality of breakfast cereals in France. BMC Public Health.

[23] Julia C, Kesse-Guyot E, Touvier M, Mejean C, Fezeu L, Hercberg S (2014). Application of the British Food Standards Agency nutrient profiling system in a French food composition database. Br JNutr.

